# *In Vitro* Biocompatibility of Hydrogel Polyvinyl Alcohol/*Moringa oleifera* Leaf Extract/Graphene Oxide for Wound Dressing

**DOI:** 10.3390/polym15020468

**Published:** 2023-01-16

**Authors:** Dwi Ratna Ningrum, Wildan Hanif, Deby Fajar Mardhian, Lia A. T. W. Asri

**Affiliations:** 1 Materials Science and Engineering Research Group, Faculty of Mechanical and Aerospace Engineering, Institute Teknologi Bandung, Jalan Ganesha 10, Bandung 40132, Indonesia; 2 Department of Dental Materials Science and Technology, Faculty of Dentistry, Universitas Padjadjaran, Sumedang 45363, Indonesia

**Keywords:** diabetic foot ulcers, *Moringa oleifera* leaves, hydrogel, polyvinyl alcohol, freeze–thaw, wound dressing

## Abstract

Hydrogel-based wound dressings are often chosen for healing diabetic foot ulcers (DFU) in combination with herbal extracts. *Moringa oleifera* leaf (MOL) extract is a potent herb containing antimicrobial and anti-inflammatory bioactive substances. In this work, wound dressings based on polyvinyl alcohol (PVA), MOL extract, and graphene oxide (GO) were developed for DFU wound dressing. The PVA/MOL/GO hydrogel was synthesized using four cycles of a freeze–thaw process with varying concentrations of MOL extract. All hydrogels showed a water content of 83–88% and an equilibrium swelling ratio between 155–171%. After degradation in phosphate-buffered saline, the hydrogels showed a more open porous structure. We observed a degradation rate of 26–28%. Although the increase in MOL extract reduced the tensile strength of the hydrogel, the addition of GO increased the tensile strength. The PVA/MOL/GO hydrogel showed the highest antibacterial activity, with a reduction of 94% Gram-positive *S. aureus* and 82% Gram-negative *E. coli*. Finally, all samples possessed appropriate cytocompatibility with cell viability reaching 83–135% in 3T3L1 mouse fibroblast cells. This result was verified by in vitro wound-healing analysis performed by scratch assay. This study presents the potency of combined PVA, MOL, and GO as a biocompatible DFU wound dressing.

## 1. Introduction

Diabetes mellitus (DM) is a chronic disease that has rapidly become a global epidemic [1]. According to the International Diabetic Federation, there were 547 million adults living with DM in 2021, which is predicted to increase to 643 million in 2030 and 783 million in 2045 [2]. Individuals with DM have an approximately 25% risk of developing ulcerations and/or destruction of the feet’s soft tissues [3]. About 20 to 33% of costs related to DM are used for the treatment of diabetic foot ulcers (DFU) [4]. Early detection and proper wound treatment can prevent amputations [5]. Treatment of early stage DFU can be performed by applying a wound dressing at the wound site, which aims to accelerate the healing process. Furthermore, this wound care is considerably more economical than other treatments [6].

Herbal medicine has an essential role in wound healing. It has been widely used since it is a natural product that can be found easily in nature and is relatively inexpensive [7]. *Moringa oleifera* leaves (MOL), for example, have been used for a long time as a traditional medicine to treat various diseases, including cancer, cardiovascular, atherosclerosis, and diabetes [8]. *Moringa oleifera* has been shown to be potent as a wound healer. One of the key components in MOL is vicenin-2, which can induce wound healing [9]. MOL also contains phytochemical class structures, including alkaloids, tannins, phenolics, steroids, saponins, and flavonoids that have several benefits, such as antioxidant, antimicrobial, anticancer, anti-inflammatory, and anti-diabetic properties [10,11,12].

Different materials have been developed as wound dressings based on the different types of wounds and how they heal. One of them is a hydrogel, which can meet the requirements for wound dressing because it can reduce pain, is simple to remove, transparent, fluid-absorbent, and capable of preventing fluid loss. The hydrogel can serve as a barrier against bacteria [13]. Hydrogel is also preferred due to its similarities to the physical properties of human tissue, which are rich in water (70–90%) and have low interfacial tension with biological fluids [14,15]. Chen et al. [16] and Khan et al. [17] reported hydrogels with hemocompatibility and antibacterial properties to accelerate wound healing.

Polymers are widely used as a matrix in wound dressing to accelerate wound healing, such as polyvinyl alcohol (PVA), polyurethane, polymethyl methacrylate, and polyvinyl pyrrolidone [18]. Currently, PVA is one of the oldest and most widely used synthetic polymer hydrogels and has been applied in several advanced biomedical applications, such as wound dressings [19]. The chemical structure of PVA is simple, and it is possible to modify it chemically using straightforward reactions. Additionally, PVA as a hydrogel is elastic and expands rapidly in biological or aqueous media. PVA has several advantages that allow it to mimic natural tissue and be completely accepted by the body [19]. However, PVA hydrogel has low mechanical strength [20]. One strategy to increase its strength and stability is by adding carbon in the form of graphene oxide (GO). GO is a derivative of graphene that has attracted much attention due to its ease of preparation in a large-scale and low-cost strategy, excellent biocompatibility, and superior mechanical properties [21]. GO-incorporated hydrogels have been reported to possess excellent mechanical properties [22]. A previous study showed that adding GO to PVA increased the tensile strength of the hydrogel by about two times higher than PVA alone, and the tensile strain value was around 400–600% [23]. In addition, GO exhibited antibacterial properties due to its ability to generate reactive oxygen species [24,25].

In this study, PVA was used as a hydrogel matrix with the addition of bioactive MOL extract to promote wound healing and antibacterial activity on the DFU wound site. Wound dressing applied to DFU should have high mechanical properties to allow high mobility. Here, we utilized GO to enhance the mechanical properties of the hydrogel. MOL- and GO-loaded PVA hydrogels were developed to work in synergy to provide better performance for, but not limited to, diabetic wound healing. By taking advantage of each material, we aimed to show their superiority when combined instead of working individually. Furthermore, to our knowledge, the hydrogel that consists of these three materials has never been reported.

We prepared the hydrogel by employing a physical crosslink method, the freeze–thaw cyclic process, to avoid the chemical crosslinker that may result in toxicity. The hydrogel’s morphology, functional group, mechanical, degradation rate, antibacterial properties, cell viability, and scratch assay were evaluated to determine its potential as wound dressing for DFU.

## 2. Materials and Methods

### 2.1. Materials

Methanol, PVA fully hydrolyzed 99% with MW 146,000–186,000 g/mol, phosphate-buffered saline (PBS, 99.9% purity), and GO 15–20 sheets (4–10% edge-oxidized), were purchased from Sigma Aldrich (St. Louis, MO, USA). All chemicals were used as received.

### 2.2. Preparation of MOL Extract

Fresh MOL from a *Moringa* plantation in Tanaman Obat Cimenyan (TAOCI Bandung, Indonesia) was used as a source of MOL extract. MOLs were cleaned by soaking them in a salt solution and rinsing them with tap water. The leaves were then dried in a clean chamber at room temperature without being exposed to direct sunlight for 4 days. The dried *Moringa* leaves were ground and then sieved to obtain a fine, uniform powder. Next, active compounds were extracted from *Moringa* leaves by the maceration method. Three-hundred-fifty grams of MOL fine powder was immersed in 1050 mL methanol for 5 days with daily stirring for ten minutes to optimize the diffusion process of bioactive compounds. The liquid was filtered through filter paper to discard the solid residue. Next, the filtered liquid was evaporated on a rotary evaporator (Buchi Rotavator R-200, Flawil, Switzerland) at 49 °C until the extract was thick and then dried in a freeze–drier (Freezone 4.5 L–Labconco, Kansas, MO, USA) to obtain the final product with a yield of 3.65%. The dried extract of MOL was stored at −20 °C for further use [9].

### 2.3. Preparation of PVA/MOL/GO Hydrogel

The hydrogels were fabricated by dissolving different concentrations of MOL (0.1 and 0.5 w/t%) in 10 w/t% PVA, with or without 0.05 w/t% GO. Ten grams of PVA powder was dissolved in 100 mL of demineralized water and stirred using a hot plate at 160 °C for 4 h until completely dissolved. The temperature was then lowered to 110 °C, and GO powder was added to the PVA solution and stirred until the PVA/GO mixture was homogeneous. The solution temperature was then lowered to 45 °C before adding MOL extract into the PVA/GO mixture. After homogenous, the mixture was allowed to stand without stirring for 2 h to remove air bubbles. Then, 20 mL of the mixture was poured into a Petri dish with a diameter of 10 cm and frozen at −20 °C for 20 h to induce crystallization. Next, PVA/MOL/GO was thawed at 5 or 25 °C for 4 h. The freeze–thaw cycle was repeated twice, and the hydrogel was obtained with an average thickness of 2.99 ± 0.13 mm.

### 2.4. Hydrogel Characterization

#### 2.4.1. Fourier-Transform Infrared (FTIR)

The presence of functional groups in the MOL extract, GO, and PVA/MOL/GO hydrogels were evaluated using FTIR. In addition, the possible formation of molecule interactions that occurred in a mixture of PVA hydrogel, MOL extract, and GO was also assessed. Analysis was performed using FTIR spectrometry (Prestige 21 Shimadzu, Tokyo, Japan). A standard detector was used in the spectrometer range of 500–4000 cm^−1^ with a resolution of 4.0 and 50 scans.

#### 2.4.2. Morphological Observations

The surface morphology of the hydrogels was observed to distinguish the differences in the hydrogels resulting from the two different thawing temperatures (5 and 25 °C). Furthermore, the morphological changes after the degradation test were also evaluated. All microscopic images were obtained by a scanning electron microscope (SEM, Hitachi SU3500, Tokyo, Japan). Prior to SEM, hydrogel specimens were freeze-dried and then cut down into 1 cm × 1 cm. The specimen was coated with gold to make it conductive using a Hitachi MC1000 Sputter (Hitachi, Tokyo, Japan).

#### 2.4.3. Water Content

Hydrogel samples (*n* = 4) with a dimension of 0.5 cm × 0.5 cm were weighed (*Wo*) and dried in an oven for 24 h. After this, the dried hydrogels were weighed again (*Wt*), and the water content was calculated using Equation (1):

(1) $$Water\;content\;\left(\%\right)=\frac{\left(Wt-Wo\right)}{Wo}\times 100$$

#### 2.4.4. Swelling Profile

The freeze-dried hydrogel samples (*n* = 3) were cut down to 0.5 cm × 0.5 cm squares and weighed to obtain the initial weight (*Wi*). Then, the samples were put into 10 mL of PBS solution at pH 7.4. The samples were rested until the designated time point for measuring the final weight (*Wf*). To ensure there was no excess water on the sample’s surface, each sample surface was wiped with a tissue. The equilibrium swelling ratio (*ESR*) value was calculated using Equation (2):

(2) $$ESR\;\left(\%\right)=\frac{\left(Wf-Wi\right)}{Wi}\times 100$$

### 2.5. Mechanical Properties

The mechanical properties of the hydrogel, such as the tensile strength and tensile strain at break, were tested using a universal testing machine (Instron 5982, Norwood, MA, USA) with a force capacity of 100 kN. The test was carried out with five samples for each group. Before the tensile test, the geometry of the sample was adjusted to the ASTM D638 type V. The tensile strength (MPa) and e (%) values were determined with a crosshead speed of 100 mm/min until the specimen fractured.

### 2.6. Degradation Test

In vitro degradation of hydrogel was performed in PBS solution (pH = 7.4; Sigma Aldrich, MO, USA) to simulate skin tissue conditions. The hydrogel was cut with a size of 1 cm × 1 cm in a square shape, and the initial weight (*Wo*) was measured. Each sample was immersed in 5 mL of PBS with time intervals (1, 2, 3, 4, 5, 6, 7, 14, and 28 days). Next, the sample was removed from the PBS, cleaned with demineralized water to remove the remaining salt, and dried with tissue paper. The final weight (*Wi*) was measured. The percentage of degradation was calculated using Equation (3) [26].

(3) $$Degradation\;\left(\%\right)=\frac{\left(Wo-Wi\right)}{Wo}\times 100$$

### 2.7. Antibacterial Activity

#### 2.7.1. Bacterial Suspension Preparation

Bacteria *Staphylococcus aureus* ATCC 6538 and *Escherichia coli* ATCC 8939 (Oxoid, Hampshire, England) were selected for antibacterial testing with the agar diffusion [27] and total plate count methods [28]. To prepare the bacterial culture, one ose of the test bacteria colony was inoculated onto a Mueller–Hinton agar plate (MHA; Oxoid, Hampshire, England). After a 24 h incubation at 37 °C, one ose from this bacterial colony was inoculated to 10 mL of Mueller–Hinton broth (MHB; Oxoid, Hampshire, England) and incubated for 24 h at 37 °C. The culture suspension was measured for absorbance at λ 625 nm according to the standard 0.5 Mc. Farland. After appropriate absorbance, the suspension was then diluted in a ratio of 1:20 in the Mueller–Hinton Broth. The decimal dilution stage of the bacterial suspension in the ratio of 1:20 was performed by taking 9 mL of 0.9% NaCl solution pipetted into a test tube. One milliliter of the bacterial test suspension (1:20) was taken and put into the first tube (10^−1^ dilution), then vortexed to homogenize. Another 1 mL was taken from the first tube, put into the second tube (10^−2^ dilution), and so on. For the dilution in the last tube as desired (10^−6^ cfu/mL), 1 mL of the result was discarded after homogenization.

#### 2.7.2. Disc Diffusion Method

The agar disc was prepared by pouring 20 mL of thawed MHA media into a Petri dish, followed by resting to solidify. To evaluate the inhibitory efficacy of the hydrogel on bacteria, *S. aureus* and *E. coli* in 0.25 mL suspension was spread on agar plates with a cotton swab. A hydrogel with a diameter of 1 cm was placed on the center surface of the MHA inocula, allowed to rest for 20 min at room temperature, then incubated at 37 °C for 24 h. After the incubation, the diameter of the inhibition zone formed around the hydrogel was measured.

#### 2.7.3. Total Plate Count Method

Another method to examine the inhibitory activity of the hydrogel is the total plate count (TPC). To analyze the reduction in bacteria colonies, 50 μL of agar under the hydrogel was diluted until a decimal dilution of 10^−8^ was obtained. The resulting 1 mL of the last dilution was then poured into a sterile Petri dish. Next, 20 mL of MHA was poured into the Petri dish and gently shaken to homogenize the suspension. After a 24 h incubation at 37 °C, the number of colonies formed was counted. The test was carried out with three samples for each group. The reduction in bacterial colonies was calculated using Equation (4):

(4) $$Bacterial\;reduction\;\left(\%\right)=\frac{\left(Control-Treated\;colony\right)}{Control}\times 100$$

### 2.8. In Vitro Biocompatibility

#### 2.8.1. Cell Culture

In vitro biocompatibility of the hydrogel samples was evaluated with 3T3L1 mouse fibroblast cells (Cell Culture and Cytogenetics Laboratory Faculty of Medicine, Padjadjaran University, Bandung, Indonesia). The cells were cultured in Dulbecco’s Modified Eagle’s Medium (DMEM, Sigma-Aldrich Corp., St. Louis, MO, USA) supplemented with 10% fetal bovine serum (FBS, Sigma-Aldrich. St. Louis, MO, USA). Cells were incubated in a humidified atmosphere containing 5% CO_2_ at 37 °C. After 24 h, the cells were washed with PBS solution and starvation cultured in DMEM–0% FBS to stop cell growth. The 3T3L1 cells were seeded into the cell culture plates containing culture medium without any hydrogel samples as the control. 

#### 2.8.2. Cytotoxicity Assay

The MTT assay was used to measure the cytotoxicity and cell viability towards mouse fibroblast 3T3L1 cells employing MTT ((3-(4,5-dimethylthiazol-2-yl)-2,5-diphenyltetrazolium bromide, Sigma-Aldrich, St. Louis, MO, USA). Samples were sterilized with UV treatment. After that, samples were incubated with DMEM for 24 h at room temperature to obtain the extract. Cells were seeded at a concentration of 5 × 10^3^ cells/well in 200 μL culture medium DMEM and various amounts of hydrogel extract (10, 50, 100 μg/mL) into 96-well microbiological plates, then incubated at 37 °C. A seeded well with no sample was used as the control. After a 24 h incubation, 100 μL of the MTT labeling reagent was added to each well and incubated at 37 °C for 4 h in a humidified atmosphere. The medium was then taken out, and 100 μL DMSO (Sigma-Aldrich, St. Louis, MO, USA) was added to stop the reaction and enable the formations of formazan crystal. Finally, the absorbance was measured using an ELISA plate reader at a wavelength of 550 nm. Cell viability was calculated by dividing the absorption of each sample by the control [29]. The values were reported as the average of five replicates.

#### 2.8.3. Wound Scratch Assay

The migration of 3T3L1 fibroblast cells, treated with different samples of hydrogel extract was performed via an in vitro wound scratch assay similar to the previous experiment [30]. The sample was sterilized by UV treatment and then immersed with DMEM for 24 h to obtain the sample extract. Simultaneously, 3T3L1 mouse fibroblast cells were cultured with a density of 15 × 10^4^ cells/well and incubated at 37 °C until confluent and a monolayer formed. A scratch was made on the cell surface with a sterile pipette and washed gently with PBS to remove the free and adhered cell from the scratched region. The obtained sample extract (50 μg/mL) with starvation (0% FBS–DMEM) was added to the scratch well and incubated at 37 °C in 5% CO_2_. Cell migration between the scratch areas were imaged at 0, 6, and 24 h using an inverted microscope Olympus CK 40 (Olympus, Tokyo, Japan).

### 2.9. Statistical Analysis

All values in the graphs are expressed as mean ± standard error of the mean (SEM). Statistical significance of these results was performed by a two-tailed unpaired Student’s *t*-test for comparison of two groups using GraphPad Prism version 5 (GraphPad Software, Inc., San Diego, CA, USA). Differences were considered significant for a *p* value of * *p* < 0.05, ** *p* < 0.01, and *** *p* < 0.001.

## 3. Results and Discussion

### 3.1. Physical Crosslink of PVA/MOL/GO Hydrogel

The freeze–thaw process was conducted to crosslink the PVA chains physically to form a gel. This physical crosslink method was selected to avoid the chemical bonding agents, which can result in toxicity [31,32]. The bioactive compounds of the MOL extract and GO acted as active agents in the hydrogel. MOL extract is used in wound healing because it contains flavonoids which are useful as antibacterial agents and vicenin-2, which can accelerate the wound-healing process [9]. GO is employed as reinforcement in the PVA hydrogel due to the orientation of the GO sheets dispersed in the hydrogel, which forms hydrogen bonds between the GO hydroxyl groups and the PVA chains [32]. The improvement of the mechanical properties of the PVA hydrogel with the addition of GO is in line with the work reported by Hanif et al., Zhao et al., and Liu et al. [23,33,34,35].

**Figure 1:**
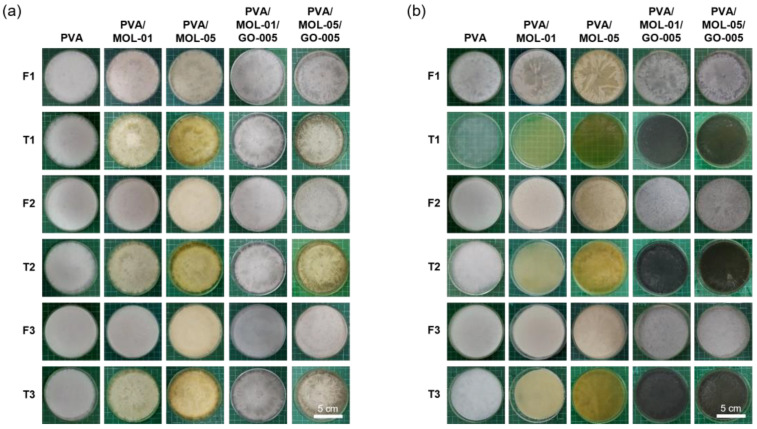
Physical appearances of hydrogels after each freezing and thawing cycle, indicated by F and T, respectively, (**a**) freezing at −20 °C and thawing at 5 °C and (**b**) freezing at −20 °C and thawing at 25 °C.

Figure 1 shows the freeze–thaw process of the hydrogel with different concentrations of MOL extract and GO and with thawing at different temperatures (5 and 25 °C). Visually, there were differences in the results after three freeze–thaw cycles. As shown in Figure 1a, hydrogels thawed at 5 °C have inhomogeneous surfaces as a white pattern was still visible. This pattern was not observed in hydrogels with a thawing temperature at 25 °C (Figure 1b). Hydrogels thawed at 5 °C did not completely melt after 4 h, most likely leading to the formation of PVA segregation represented by emerging white layer on the surface. 

In contrast, a faster thawing rate at 25 °C might have prevented segregation due to phase separation. There was a difference between the PVA-rich phase and the water-rich phase. The phase separation in the hydrogels thawed at 5 °C was because the PVA chains cannot completely rearrange themselves, resulting in few areas rich in crystallites. The crystallite area increased as the freeze–thaw cycles increased and accumulated in the same section, resulting in a phase separation rich in the PVA phase. Phase separation is a unique mechanism that occurs during the PVA gelation in the freeze–thaw process. Several studies have reported the presence of phase separation [36,37,38]. This phase separation phenomenon will impact the properties of the hydrogel material applied to biomedicine, especially its mechanical properties [39].

### 3.2. Characterizations

**Figure 2:**
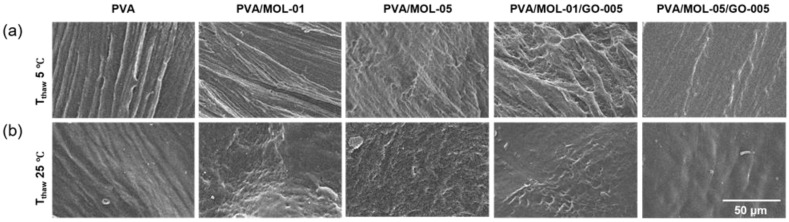
Surface morphology of freeze–thawed hydrogels prepared at two different thawing temperatures: (**a**) 5 °C and (**b**) 25 °C.

The SEM images showed morphological differences in the presence of phase separation. In Figure 2a, the hydrogels prepared via the 5 °C thawing process show a morphology that is rather wavy and porous. On the other hand, the surface of the hydrogels thawed at 25 °C (Figure 2b) tend to be flat and contain more pores. This result is in line with previous studies [39,40,41] that explained the presence of pores in the hydrogel after the freeze–thaw cycle. The presence of pores could facilitate the growth and migration of dermal fibroblasts, accelerating tissue healing allowing oxygen permeation to maintain a moist wound environment, and be useful for entry and exit routes for water and active substances, thereby creating conditions for its application as a wound dressing [42]. Therefore, hydrogels with a 25 °C thawing process were further evaluated in this study.

**Figure 3:**
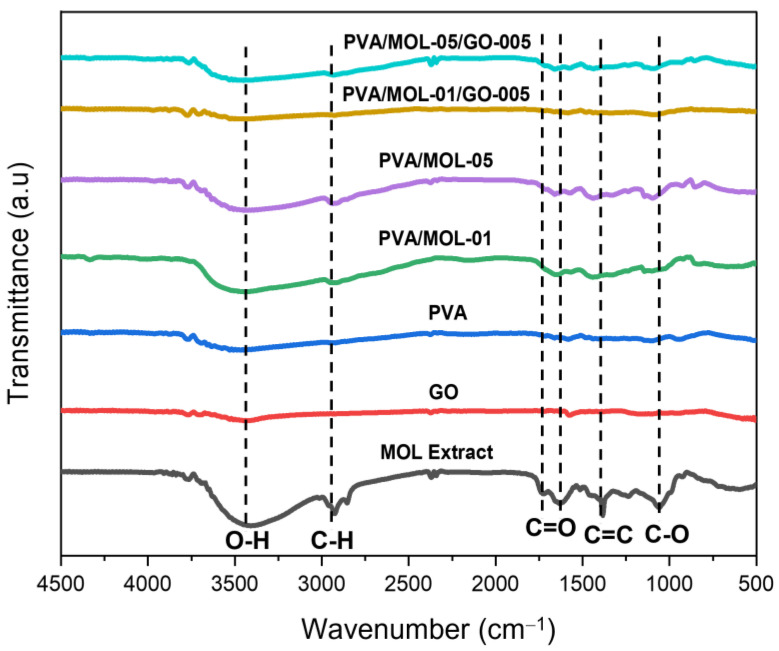
FTIR spectra of constituents of the hydrogels and the different hydrogels as indicated.

FTIR spectra confirmed the presence of PVA, MOL extract, and GO in hydrogels respective to their components. Figure 3 shows the peak of MOL extract of the O-H stretching vibrations at 3408 cm^−1^ and the C-H stretching vibrations at 2926–2854 cm^−1^. Carbonyl group (C=O) peaks were found at 1633 cm^−1^, while stretching vibrations (C=C) of aromatic rings and C-O peaks were observed at 1382 and 1058 cm^−1^, respectively. The hydroxyl groups contained in the extract were largely found in polyphenol molecules such as tannins, flavonoids, and glycoside derivatives. All peaks on the MOL extract showed the presence of phenolic structures [43,44]. Strong absorption at 3450 cm^−1^ of GO indicated O-H group stretching vibrations. A peak at 1575 cm^−1^ showed C=O stretching vibrations [45], while C-O stretching vibrations were highlighted by peaks at 1166 and 1024 cm^−1^, which indicated carboxylic acid. The oxygen-containing group of GO revealed that the graphene was oxidized. The hydroxyl group as the polar group resulted in the formation of hydrogen bonds and indicated that GO is hydrophilic [46]. Meanwhile, the PVA spectrum showed the characteristics of the hydroxyl group at 3468 cm^−1^ (O-H stretching vibrations), 2937 cm^−1^ (C-H stretching vibrations), 1722–1660 cm^−1^ (C=O stretching vibrations), and 1095 cm^−1^ (C-O stretching vibrations) [14].

Compared to the FTIR spectra of the main materials, the hydrogel, with the addition of MOL extract and GO, produced a spectrum that was a combination of the main constituent materials. All hydrogel samples showed the strongest peaks among the others in the hydroxyl group (O-H stretching vibration) at 3427–3448 cm^−1^ and alkyl (C-H stretching vibration) at 2920–2937 cm^−1^. Hydrogen bonds between OH groups occurred between PVA chains and MOL extract or GO [14].

**Figure 4:**
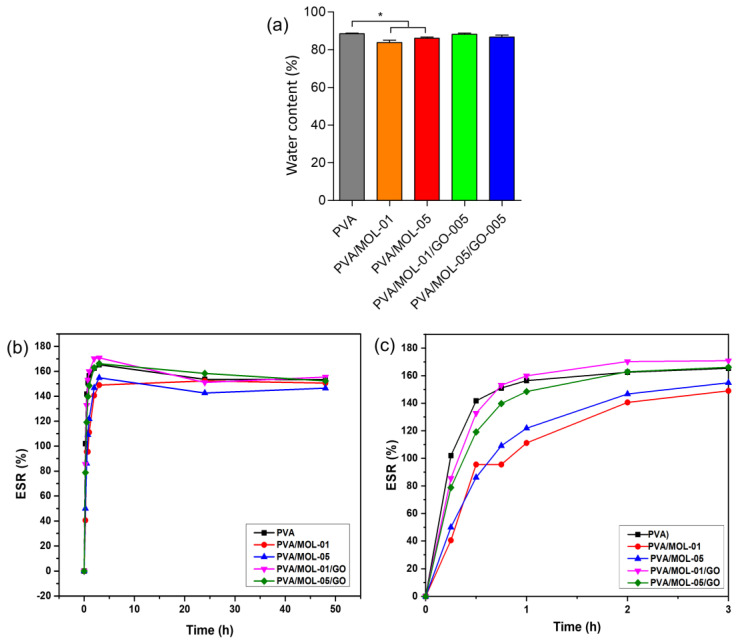
(**a**) Water content. (**b**) Swelling profile of all the hydrogels time vs. ESR at timepoint 0–48 h and (**c**) 0–3 h. Data represents means ± SD from at least three independent experiments. * *p* < 0.05.

Water content measurements indicated the hydrophilic properties of the hydrogels. All hydrogels showed a high-water content, between 83.75–88.45% (Figure 4a). The hydrophilic nature of the hydrogels is favorable for facilitating the interaction with the surrounding tissue at a wound site. 

We used PBS with a pH of 7.4 as the test environment to mimic the body fluid conditions in terms of osmolality. The equilibrium swelling ratio (ESR) value of the hydrogels increased over time (Figure 4b). The hydrogel samples swelled easily because of the surface’s hydrophilic properties. Initially, PVA/MOL-01 and PVA/MOL-05 showed lower ESR values than other samples (Figure 4c). They then exhibited a similar profile reaching a plateau after 24 and 48 h. After 15 min of incubation, the ESR value of PVA hydrogel was about 102%, while PVA/MOL-01 and PVA/MOL-5 only reached 41% and 50%, respectively. The addition of GO into the hydrogel increased the ESR value to 86% and 75% for PVA/MOL-01/GO-005 and PVA/MOL-05/GO-005, respectively. The lower the ESR value shown by the hydrogels containing MOL and GO might be due to a denser hydrogel structure than PVA. MOL and GO components can be entrapped in the PVA matrix and fill the pores produced by PVA, causing the solvent to be more difficult to penetrate through the hydrogel in the beginning stages of swelling. All samples demonstrated the highest ESR value after 3 h of incubation, with 165%, 155%, 168%, 171%, and 170% for PVA, PVA/MOL-01, PVA/MOL-05, PVA/MOL-01/GO-005 and PVA/MOL-05/GO-05, respectively. These values slightly decreased after 24 h, indicating the release of some components from the hydrogels. After 48 h, the ESR values were almost similar for all samples, with 153%, 151%, 147%, 155% and 152% for PVA, PVA/MOL-01, PVA/MOL-05, PVA/MOL-01/GO-005 and PVA/MOL-05/GO-05, respectively. 

### 3.3. Mechanical Properties

**Figure 5:**
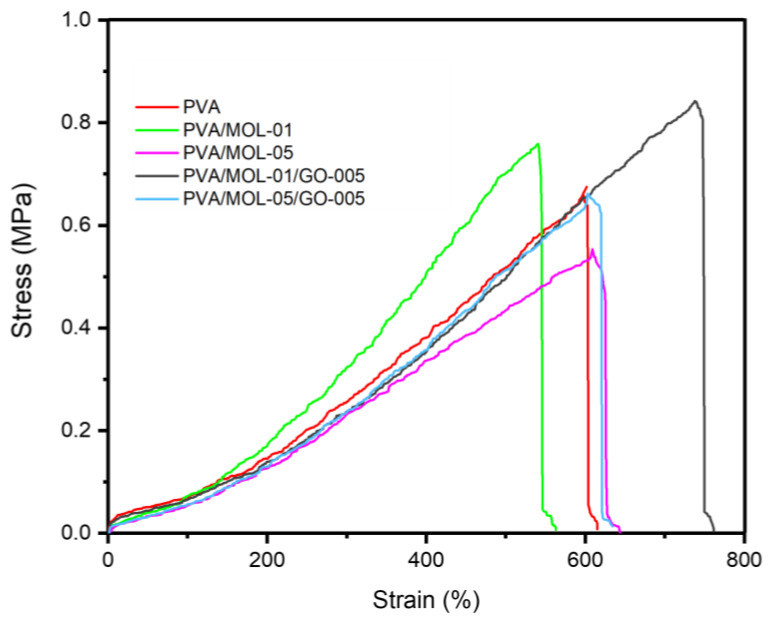
Stress–strain curve of all the hydrogels.

The mechanical properties of the hydrogels have a critical role in their function as a wound dressing for DFU. Ideally, a wound dressing is flexible and durable and can cover the wound fitting its position [47]. This effect of MOL extract and GO on the PVA hydrogel is displayed in Figure 5. PVA hydrogel has a tensile strength of 0.550 MPa and a tensile strain of 537.94%, as shown in Table 1. Adding 0.1 w/t% MOL extract reduced flexibility, i.e., an increase in tensile strength to 0.617 MPa and a strain reduction to 497.48%. However, the higher concentration of MOL extract (0.5 w/t%) reduced the tensile strength to 0.463 MPa with a relatively similar strain. This effect of higher MOL extract incorporation into the hydrogel was due to weaker hydrogen interactions, as MOL substances hindered the interactions between the PVA hydroxyl groups. This result is in line with works conducted by Naqvi et al. [48] and Ibrahim et al. [49].

As expected, adding GO to the hydrogel increased the tensile strength to 0.677 MPa with an ultimate strain of 684.23% in PVA/MOL-01. A similar effect was also observed in PVA/MOL-05/GO-005, increasing the tensile strength to 0.547 MPa from 0.463 MPa (PVA/MOL-05). This phenomenon is due to the interaction between the PVA chains and the GO nanosheets. The hydroxyl and carboxyl functional groups from the basal plane and the GO edge form strong hydrogen interactions with the hydroxyl groups in PVA [50], which provides a better load transfer between PVA and GO to improve the mechanical properties [51]. During the tensile process, GO sheets are aligned along the stretching direction due to the hydrogen bonds between the hydroxyl and carboxyl groups in GO and the PVA chain. Therefore, when the hydrogel is stretched, the GO sheets are also stretched, which increases the breaking elongation [50].

Regarding the ability to stretch, the hydrogel showed excellent value, represented by a tensile strain value of more than 400%. A previous study showed that a PVA hydrogel combined with herbs had a tensile strength of 0.5–2 MPa with elongation of 200–300% [48]. This property is crucial to ensure flexibility in the skin contours. More importantly, the value of this property in the hydrogel is similar to skin so that it can be applied effectively to wounds [52].

### 3.4. Degradation Test

**Figure 6:**
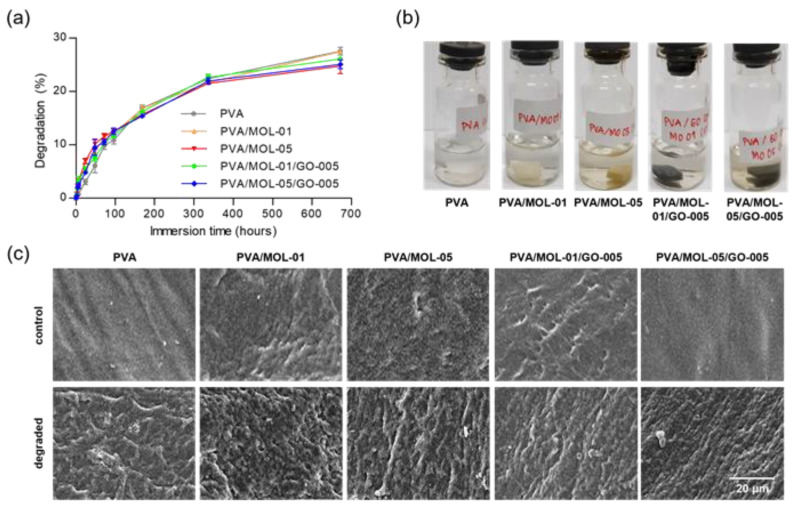
(**a**) Degradation of the hydrogels in PBS. (**b**) Representative images of the liquid in which the degradation test was performed. (**c**) Surface morphology before degradation (control) and after 336 h of immersion in PBS (degraded).

Another crucial property of hydrogels as wound dressings for wound healing is their degradation properties. The hydrogel degradation rate indirectly affects cell function and host tissue remodeling [53]. Figure 6a shows that the degradations of all the prepared hydrogels were mostly similar. Each hydrogel sample showed around 26–28% weight loss after 28 days, indicating a slow degradation rate that provides sufficient time for skin regeneration [54]. The hydrogels were degraded due to the dissolution of PVA in all samples. The hydrogen interaction between the PVA chains became tenuous and broken. After 28 days of immersion, the hydrogels with MOL extract showed discoloration in the PBS, most likely caused by the release of the MOL extract (Figure 6b).

The degradation was also shown by the changes in the surface morphology of each hydrogel (Figure 6c). There was a significant change in the surface morphology after 14 days of immersion, and more open pores were observed on the surface. The hydrogels containing MOL extract (PVA/MOL-01 and PVA/MOL-05) had more open pores than the ones with GO. The open pores are attributed to the high solubility of PVA and MOL extract in water. In line with previous studies [55,56], as PVA and MOL extract starts to dissolve in water, the voids facilitate the degradation, and more open pores are formed. The results obtained by the presence of pores have met the principles of wound dressings, to create a moist environment around them due to oxygen permeation [57].

### 3.5. Antibacterial Activity Test

Wound dressings must possess antibacterial properties since bacteria are the main problem affecting the wound healing process, especially for DFU wounds. Threats of infection, contamination, or bacterial colonization must be adequately addressed. Wound dressings with antibacterial properties can stop microbial colonization and prevent infection [58].

Figure S1 shows that the agar diffusion method was unsuitable for evaluating the hydrogels’ antibacterial activity. The agar diffusion was performed to qualitatively evaluate the antibacterial activity of the hydrogels. We employed Gram-positive bacteria *S. aureus* ATCC 6538 and Gram-negative *E. coli* ATCC 8939 in the test. Only the MOL extract and GO alone showed clear zones of 17.1 mm and 11.0 mm, respectively, against *S. aureus* bacteria. Meanwhile, no zone of inhibition was observed on agar with *E. coli*. This is most likely caused by the limited diffusion of MOL extract and GO in the hydrogels [59]. Thus, we evaluated the antibacterial properties of the hydrogels further using the total plate count (TPC) method.

**Figure 7:**
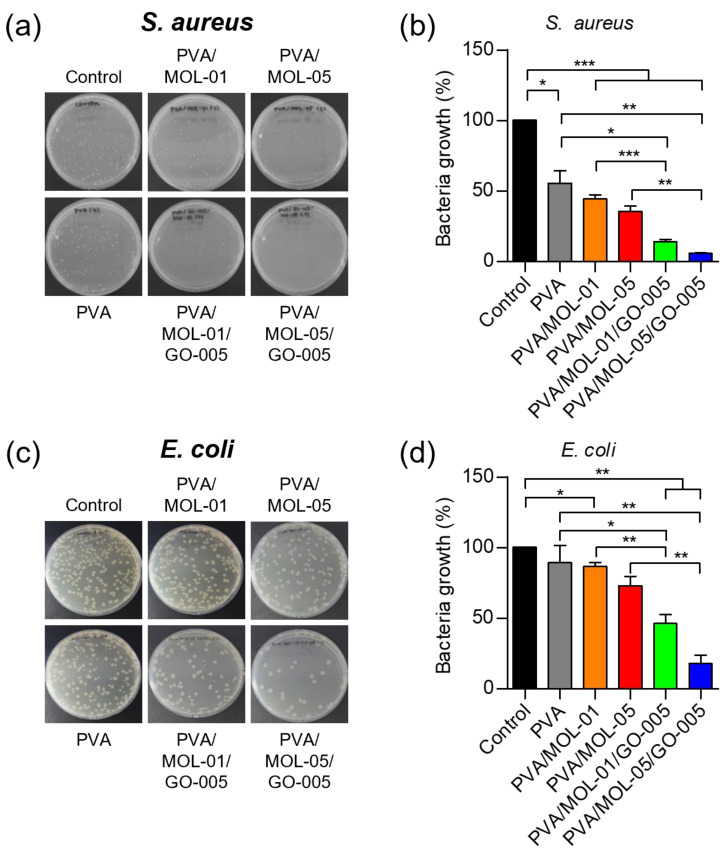
Antibacterial activity of hydrogels towards *S. aureus* ATCC 6538 and *E. coli* ATCC 8939 using the TPC method after a 24 h incubation. (**a**) Representative images and (**b**) quantitative analyses of *S. aureus* colony growth treated with the different hydrogels. (**c**) Representative images and (**d**) quantitative analyses of *E. coli* colony growth treated with the different hydrogels. Data represents means ± SD from at least three independent experiments. * *p* < 0.05, ** *p* < 0.01, *** *p* < 0.001.

Using the TPC method, the hydrogels showed antibacterial activity against both *S. aureus* and *E. coli*. As shown in Figure 7a,b, the hydrogels containing MOL inhibited the growth of *S. aureus*, and a higher concentration of MOL provided a higher efficacy. In addition to increasing the strength, GO in the hydrogels also enhanced the antibacterial effect, resulting in almost complete inhibition (94% bacterial reduction) in cultures treated with PVA/MOL-05/GO-005. A similar trend of inhibitory effect was observed in the evaluation against *E. coli*, although at a lower inhibition (82% bacterial reduction) due to the characteristics of Gram-negative bacteria (Figure 7c,d).

The difference in the inhibitory effect of the hydrogels against Gram-positive and Gram-negative bacteria can be explained by the actions of cell membrane penetration. Gram-positive bacterial cell walls contain peptidoglycan, the essential component of the cell membrane. The critical attack site of anti-cell-wall agents is the peptidoglycan layer. This layer is essential for the survival of bacteria in hypotonic environments; loss or damage of this layer destroys the rigidity of the bacterial cell wall [60]. The antibacterial properties of MOL and GO can damage the cell membrane, which consists of the main components of peptidoglycan, causing a loss of cell permeability and cell death in *S. aureus* bacteria. 

On the other hand, Gram-negative bacteria, *E. coli*, apart from having cytoplasmic cell membranes, also possess an outer membrane regulated by β-barrel proteins in the porin lining. Thus, transport through the cell membrane is more complex, resulting in a low outer membrane permeability and hence providing a high intrinsic antibacterial resistance [61]. 

The antibacterial properties of the MOL extract are contributed by alkaloids and flavonoids that influence pathogenic bacteria. Furthermore, its antibacterial properties are associated with the presence of gallic acid, tannins, saponins, and phenolic compounds such as alkaloids and flavonoids, which inhibit bacterial proliferation [62]. 

GO contributed not only to the hydrogel’s physical strength but also its antibacterial effect. A major cause of its toxicity to bacteria is the oxidative stress that generates reactive oxygen species (ROS) [63], leading to the deactivation of proteins and lipids, which are essential in proliferation [64]. In addition to oxidative stress, GO can physically kill bacteria by cutting the cell membrane with its sharp edges, leading to leakage and cell death. Another mechanism of GO’s antibacterial properties is wrapping bacteria and isolating them from nutrients, which significantly reduces their proliferation [65]. Altogether, the antibacterial properties of MOL extract and GO inhibit the bacteria population and can be developed as an optimal antibacterial agent to kill bacteria in its applications as a wound dressing.

### 3.6. Biocompatibility Study

**Figure 8:**
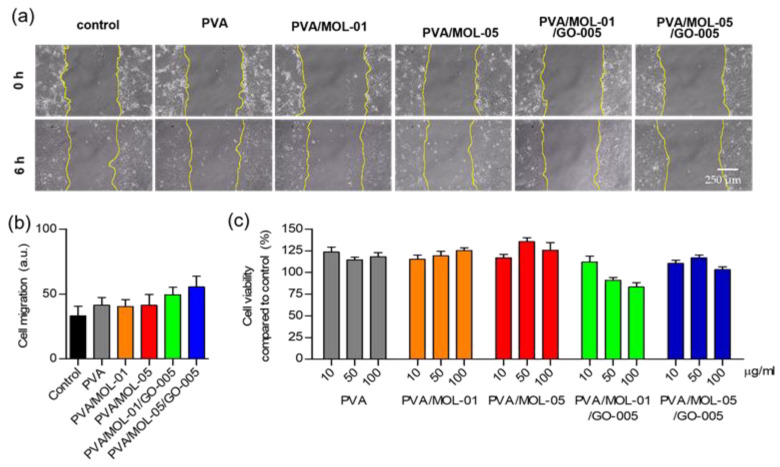
In vitro evaluation of cell migration and cytotoxicity using the mouse fibroblast 3T3L1 cells line. (**a**) Representative microscopic image and (**b**) quantitative analysis of the cell migration assay measured by the gap of the scratch area after a 6 h incubation at 50 μg/mL concentration. (**c**) Cytotoxicity assay cultured on the hydrogels after a 24 h incubation compared to control treated with different concentrations. Data represents means ± SD from at least five independent experiments.

The biocompatibility of the hydrogels was assessed with 3T3L1 mouse fibroblast cells to determine the hydrogel’s safety for host tissues using wound scratch and MTT assays. The scratch assay was used to investigate in vitro wound healing based on cell migration and growth for the regeneration of a new dermal structure [66]. Quantification of cell migration for all samples was analyzed using ImageJ [67]. Figure 8a shows images of the scratched areas captured at 0 and 6 h. The cells gradually migrated to the scratched area over time. Figure 8b shows that PVA/MOL-05/GO-005 had the highest cell migration after 6 h. Quantified analysis showed that the hydrogels with the addition of MOL extract and GO showed no reduction in migratory activity. The gaps were completely recovered after 24 h (Figure S2), indicating the hydrogels’ biocompatibility. The presence of GO favored the adhesion and spread of cells which accelerated the gap closure [68].

The cytotoxicity of hydrogels was evaluated by an MTT assay. In Figure 8c, the viability of all the hydrogel extracts with concentrations 10, 50, and 100 μg/mL showed no sign of a toxic impact on the cells with the range of viability between 83–135%. According to ISO 10993–5:2009 guidelines, the viability of more than a 30% reduction in cells is considered a cytotoxic effect [69]. Our results indicate that the hydrogels were considered non-cytotoxic. All hydrogel samples maintained the cell viability above 100% except for PVA/MOL-01/GO-005, which had 91% (50 μg/mL) and 83% (100 μg/mL) after 24 h of incubation. On the other hand, the hydrogel of PVA/MOL-05 with 50 μg/mL concentration demonstrated the highest cell viability of 135%, in comparison with other prepared hydrogels. It seems that the addition of the MOL extract to the hydrogel increased the cell viability.

## 4. Conclusions

We have reported the formulation of PVA hydrogels containing GO and MOL extract using the freeze–thaw method. GO plays a role in reinforcing the hydrogels, improving the tensile properties of the hydrogels. The addition of MOL extract was aimed to improve the biocompatibility and wound healing. The PVA hydrogels containing MOL and GO showed a high water content and a relatively low degradation rate. The surface morphology of the hydrogels immersed in PBS demonstrated more open pores, which may facilitate fibroblast adhesion and proliferation. Biologically, the hydrogels showed antibacterial activity, enhanced with the increasing concentration of MOL and the addition of GO against *S. aureus* and *E. coli*. According to the scratch assays, the hydrogels could enhance the in vitro wound-healing efficiency due to appropriate cell growth and migration. Hydrogels with the MOL extract and GO showed no reduction in the migratory activity of 3T3L1 mouse fibroblast cells. Finally, the study of the toxicity of hydrogels indicated that the hydrogels were cytocompatibility with fibroblast cells, with a viability around 83–135%. The addition of the MOL extract to a hydrogel improves cell viability. Therefore, the results conclude that the PVA/MOL/GO hydrogel can be a promising biomaterial for applications in wound dressings.

## Figures and Tables

**Table 1:** Data of the PVA/MOL/GO hydrogel tensile strength and stain.

Sample	Tensile Strength (MPa)	Tensile Strain (%)	Young’s Modulus (MPa)
PVA	0.550 ± 0.108	537.94 ± 55.30	0.142 ± 0.019
PVA/MOL-01	0.617 ± 0.125	497.43 ± 56.02	0.187 ± 0.039
PVA/MOL-05	0.463 ± 0.081	539.05 ± 61.58	0.118 ± 0.014
PVA/MOL-01/GO-005	0.677 ± 0.148	684.23 ± 46.13	0.137 ± 0.018
PVA/MOL-05/GO-005	0.547 ± 0.121	531.64 ± 75.94	0.159 ± 0.007

## Data Availability

Not applicable.

## Supplementary Materials

The following supporting information can be downloaded at: https://www.mdpi.com/article/10.3390/polym15020468/s1, Figure S1: Inhibition zone of hydrogel samples using disc diffusion method with 24 hours incubation against Gram-positive bacteria; Figure S2: Cell migration assay of mouse fibroblast 3T3L1 cells on various hydrogel surfaces that showed complete closure of scratch areas after 24 h of incubation.
