# Supplementary material for: In Vitro Biocompatibility of Hydrogel Polyvinyl Alcohol/Moringa oleifera Leaf Extract/Graphene Oxide for Wound Dressing
Source: Polymers (Basel). 2023 Jan 16;15(2):468. doi: 10.3390/polym15020468 (PMC9864398; doi:10.3390/polym15020468)
Supplement: Supplementary file 1 [file polymers-15-00468-s001.zip › polymers-2089817-supplementary.pdf]

Supplementary Materials

In vitro biocompatibility of hydrogel polyvinyl alcohol/moringa oleifera leaves extract/graphene oxide

Dwi Ratna Ningrum[1], Wildan Hanif[1], Deby Fajar Mardhian[2], Lia A.T.W. Asri[1]*

[1]Materials Science and Engineering Research Group, Faculty of Mechanical and Aerospace Engineering, Institut Teknologi Bandung, Jalan Ganesha 10, Bandung 40132, Indonesia

[2]Department of Dental Materials Science and Technology, Faculty of Dentistry, Universitas Padjadjaran, Sumedang 45363, Indonesia

*lia.asri@material.itb.ac.id

S1. Inhibition Zone

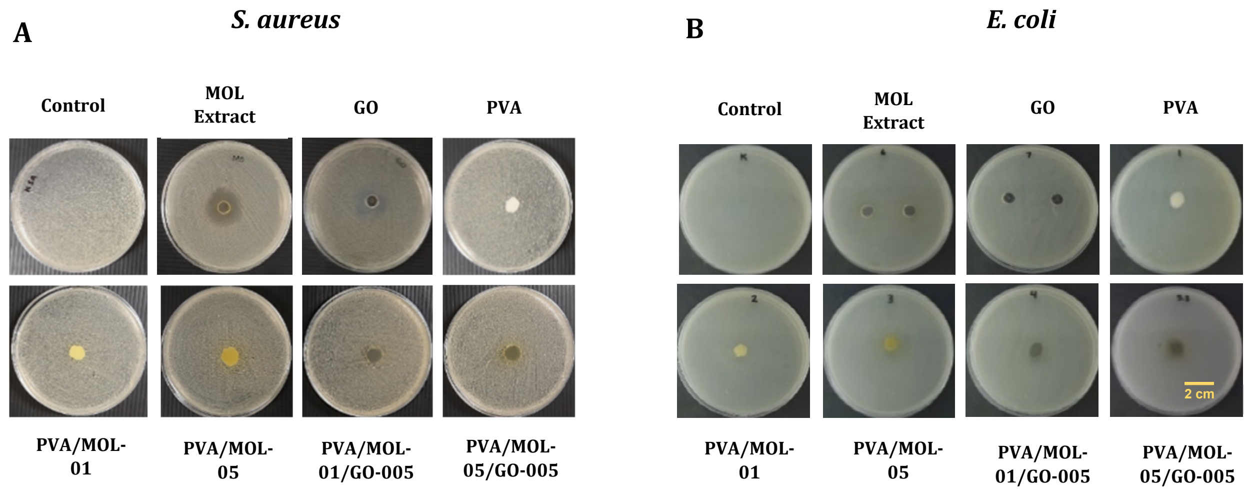

Figure S1. Inhibition zone of hydrogel samples using disc diffusion method with 24 hours incubation against Gram-positive bacteria (A) Staphylococcus aureus ATCC 6538 and Gram-negative bacteria (B) Escherichia coli ATCC 8939. MOL extract and GO showed inhibition zone and all hydrogels only showed clear zone under samples.

S2. Cell Migration Assay

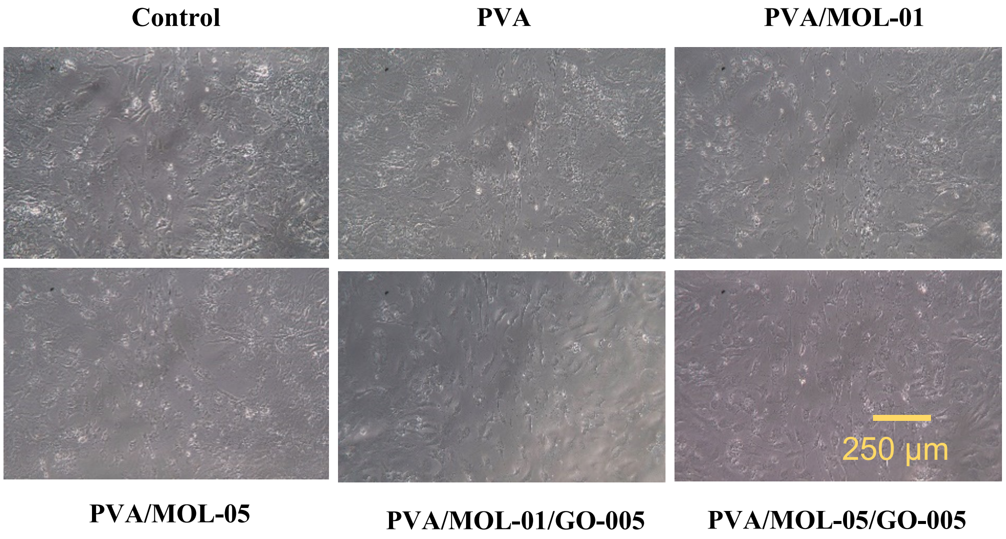

Figure S2. Cell migration assay of mouse fibroblast 3T3L1 cells on various hydrogel surfaces that showed complete closure of scratch areas after 24 h of incubation.
